# Analysis on the clinical features of 22 basaloid squamous cell carcinoma of the lung

**DOI:** 10.1186/1749-8090-6-10

**Published:** 2011-01-26

**Authors:** Li C Wang, Lei Wang, Sabrina Kwauk, Jennifer A Woo, Li Q Wu, Hong Zhu, Li Z Zhan, Na L Sun, Lei Zhang

**Affiliations:** 1 Department of Thoracic Surgery, Tianjin Lung Cancer Center, Tianjin Medical University Cancer Institute and Hospital, Tianjin, PR China; 2 School of Public Health, Harvard University, Boston, Cambridge, USA; 3 Department of Pathology, Tianjin Lung Cancer Center, Tianjin Medical University Cancer Institute and Hospital, Tianjin, PR China; 4 Tianjin Medical University, Tianjin, PR China; 5 Georgetown University School of Medicine, Washington DC, USA

## Abstract

**Background:**

Basaloid squamous cell carcinoma of the lung is a rare and highly malignant tumor mostly observed in the proximal bronchi. Basaloid squamous cell carcinoma of the lung cases typically show rapid clinical progression, very poor prognosis and special pathological morphology. This project aimed to examine the clinical features of basaloid squamous cell carcinoma of the lung and the factors related to its prognosis; and to compare survival outcomes between basaloid squamous cell carcinoma and poorly differentiated squamous cell carcinomas (PDSC).

**Methods:**

Between January 2004 and December 2008, pathological sections from basaloid squamous cell carcinoma and PDSC of the lung were collected and retrospectively analyzed at Tianjin Medical University Cancer Institute and Hospital. Data analysis was performed using Statistical Package for the Social Sciences (SPSS11.0). The Kaplan-Meier method was used to calculate the survival rate. Log-rank test was used to compare the differences in survival rate between the two groups. The factors influencing prognosis were analyzed using the Cox proportional hazard model.

**Results:**

A total of 120 pathological sections were used in the analysis of this study-22 from basaloid squamous cell carcinoma cases and 98 from PDSC cases. Compared to the PDSC group, the basaloid squamous cell carcinoma group had a larger proportion of female patients (p = 0.001); however it had higher proportion of male smokers (p = 0.003). There were no statistically significant differences in survival rate between the two groups (χ^2 ^= 1.200, p = 0.273). Additionally, prognosis of basaloid squamous cell carcinoma is significantly influenced by treatment mode and clinical stages of the tumor. The post-operation mortality hazard of patients treated with a combination chemotherapy and radiotherapy was 1.296 times higher than other treatment modes (*p *= 0.025). Increases in post-operation mortality hazard ratio were also associated with more advanced clinical stage of tumors (χ^2 ^trend = 11.907, *p *= 0.000).

**Conclusions:**

This study demonstrated that basaloid squamous cell carcinoma and PDSC have very similar clinical features, and there are no significant differences in survival rates between the two groups. Hence, we conclude that in the short term, the same clinical treatments and therapeutic modes can be administered to patients with basaloid squamous cell carcinoma and PDSC of the lung.

## Background

Basaloid carcinoma of the lung is a rare, highly malignant and aggressive lung tumor with a high rate of metastasis and death [1-3]. Basaloid carcinoma of the lung was first described in a study conducted by Brambilla in 1992, which analyzed the ultrastructural features of basaloid carcinoma of the lung [4]. Of the 38 cases analyzed, basaloid carcinoma of the lung was pure in 19 cases, and had a well-differentiated squamous cell carcinoma component with intercellular bridging and individual cell keratinization in 10 cases [4]. In 1999, the World Health Organization (WHO) and International Association for the Study of Lung Cancer (IASLC) defined basaloid carcinoma as a variant of either squamous cell carcinoma or large cell carcinoma [5]. Basaloid squamous cell carcinoma of the lung cases typically show rapid clinical progression, very poor prognosis and special pathological morphology. So it has attracted the attention of many scholars. Through retrospectively analyzing cases of basaloid squamous cell carcinoma of the lung and PDSC, this study examines the clinical features of basaloid squamous cell carcinoma to determine whether independent clinical treatment is needed.

## Methods

Between January 2004 and December 2008, pathological sections from basaloid squamous cell carcinoma and PDSC of the lung were collected and retrospectively analyzed at Tianjin Medical University Cancer Institute and Hospital. Basaloid carcinoma diagnosis was based on four criteria: (1) Invasive finger-like growth of a solid lobular or anastomotic trabecular pattern from the bronchial and/or glandular duct lining; (2) Small cuboidal to fusiform cells with a mean diameter of 12-15 μm, moderately hyperchromatic nuclei, and no prominent nucleoli (there may be a scant nucleoli with visible cytoplasm and no nuclear molding); (3) Peripheral palisading with radially arranged cells at the periphery of lobules; and (4) A high rate of mitosis between 15-44 per 10 high-power fields [4]. Additionally, basaloid squamous cell carcinoma of the lung pathological diagnosis was based on the criteria defined by Brambilla et al.: squamous cell differentiation or intercellular bridging and individual cell keratinization can be seen within the basal cell component, and the squamous cell component takes up less than half of the basal cell component [4].

Clinical features including patient gender, age and smoking history, clinical and pathological stage of the tumor, treatment modalities, and survival status were collected for each case. The clinical stage of tumors was determined using the IASLC's tumor, node, and metastasis (TNM) classification for lung cancer (the 7^th ^edition). Survival time was measured from the date of surgery to the last month of follow-up clinical exam/telephone call (April 2010 is the deadline) or death. Data analysis was performed using Statistical Package for the Social Sciences (SPSS11.0). Survival rates of the basaloid squamous cell carcinoma and PDSC groups were calculated using the Kaplan-Meier method and differences in the survival rates between the two groups were compared using the Log-rank test. In addition, the factors influencing prognosis were analyzed using the Cox proportional hazard model. Hazard ratios and 95% confidence intervals (95% CI) were calculated, and a *p*-value of <0.05 was considered to be statistically significant.

## Results

A total of 121 pathological sections were retrospectively analyzed-19 from basaloid squamous cell carcinoma cases and 102 from PDSC cases. One of the 19 basaloid squamous cell carcinoma sections had a pure basaloid pattern and was reclassified as a variant of a large cell carcinoma. Additionally, of the 102 PDSC sections, four sections displayed features that were in line with the diagnostic criteria of basaloid squamous cell carcinoma of the lung, and were reclassified as basaloid squamous cell carcinoma. Consequently, a total of 120 pathological sections are used in the analysis of this study-22 from basaloid squamous cell carcinoma cases and 98 from PDSC cases.

Clinical features of basaloid squamous cell carcinoma and PDSC cases are summarized in Table 1. Compared to the basaloid squamous cell carcinoma group, the PDSC group had a significantly higher proportion of male patients (p = 0.001). There was also a significant difference in the proportion of male smokers between the two groups (p = 0.003), but not in the proportion of both male and female smokers (p = 0.513).

**Table 1:** The comparison of clinical features between 22 cases of basaloid squamous cell carcinoma and 98 cases of PDSC

Variable	basaloid squamous cell carcinoma (%)	PDSC (%)	*p *value
Sex (male/female)	14(63.6)/8(36.4)	91(92.9)/7(7.1)	0.001
Mean age (years)	58.6	60.5	0.363
Smoking history	18(81.8)	85(86.7)	0.513
Packs/year	26.3	20.8	0.103
Packs/year (male)	33.0	20.5	0.003
Cardiovascular history	6(27.3)	18(18.4)	0.516
Peri-operative death	0(0.0)	4(4.1)	1.000
Post-operative stages			0.101
I	9(40.9)	55(56.1)	
II	8(36.4)	16(16.3)	
III	4(18.2)	26(26.5)	
IV	1(4.5)	1(1.0)	
Operation types			0.487
Lobectomy	16(72.7)	67(68.4)	
Segmentectomy	3(13.6)	8(8.2)	
Pneumonectomy	3(13.6)	23(23.5)	
Post-operative treatment			0.448
Chemotherapy	7(31.8)	33(33.7)	
Radiation therapy	1(4.5)	7(7..1)	
Combined	1(4.5)	15(15.3)	
None	13(59.1)	43(43.9)	

PDSC = poorly differentiated squamous cell carcinomas

**Figure 1:**
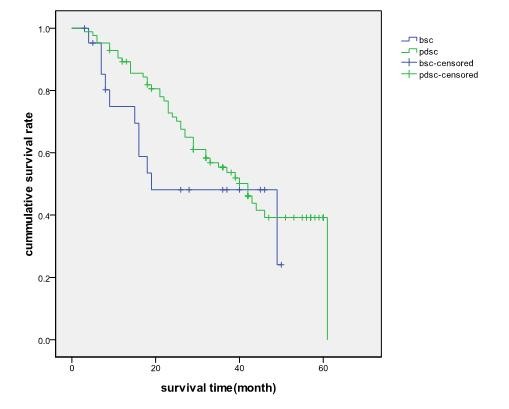
The actual survival time of basaloid squamous cell carcinoma and PDSC patients in stage I-IV, p = 0.273

**Figure 2:**
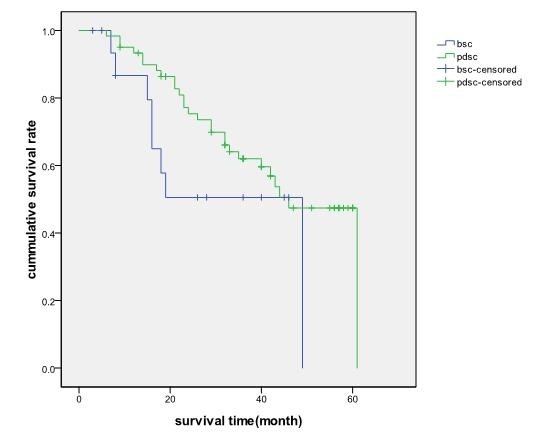
The actual survival time of basaloid squamous cell carcinoma and PDSC patients in stage I and stage II, p = 0.145

Among the basaloid squamous cell carcinoma cases, all 22 follow ups were completed. Eleven deaths were reported over an average follow up period of 22 months. Among the PDSC group, thirteen cases failed to be followed up, 45 deaths were reported over an average follow up period of 30 months-44 patients died of lung cancer and 1 patient died of heart failure. The median survival time of basaloid squamous cell carcinoma and PDSC cases were 19 and 42 months respectively. As shown in figure 1, there were no significant differences in survival rates of patients in stage I-IV between basaloid squamous cell carcinoma and PDSC cases (χ^2 ^= 1.200, *p *= 0.273). Furthermore, there were no significant differences in survival rates of patients in stage I and II between the two groups, as shown in figure 2.

Lastly, it was found that the prognosis of patients was significantly influenced by treatment mode and clinical stage of the tumor. The post-operation mortality hazard of patients treated with a combination of chemotherapy and radiation therapy was 1.296 times higher than other treatment modes (p = 0.025). In addition, the post-operation mortality hazard of patients increased with advanced clinical stage, which is summarized in Table 2 (χ^2 ^trend = 11.907, *p *= 0.000). The post-operation mortality hazard of patients in stage III and IV was 2.035 times greater than patients in stage I (p = 0.047). There were no statistical differences in post-operation mortality hazard between patients in stage I and stage II (hazard ratio 2.006, p = 0.069). Furthermore, no statistical associations were found between the prognosis of patients and their age, gender, smoking status, history of cardiovascular disease, pathological type (i.e. basaloid squamous cell carcinoma or PDSC), and type of post-operation treatment.

**Table 2:** The influence factors of post-operative survival Cox proportional hazard model analysis

Variable						95.0% CI used for Exp(B)	
	B	SE	Wald	*p *value	Hazard ratio	low	high
basaloid squamous cell carcinoma/PDSC	-.486	.382	1.618	.203	.615	.291	1.301
age ≥ 70	.668	.375	3.173	.075	1.950	.935	4.064
sex	.166	.517	.103	.748	1.181	.429	3.252
Smoking	-.253	.367	.476	.490	.776	.378	1.593
Cardiovascular disease	.408	.348	1.374	.241	1.503	.760	2.971
Operative type-Lobectomy			5.293	.071			
Operative type-Segmentectomy	-.562	.378	2.209	.137	.570	.272	1.196
Operative type-Pneumonectomy	.440	.545	.653	.419	1.553	.534	4.519
Left/right lung	.108	.315	.119	.730	1.115	.602	2.065
Clinical stage-stage I			4.837	.089			
Clinical stage-stage II	.696	.383	3.299	.069	2.006	.946	4.250
Clinical stage-stage III/IV	.710	.358	3.929	.047	2.035	1.008	4.107
Combined therapy	.831	.371	5.013	.025	2.296	1.109	4.751

CI = confidence intervals

PDSC = poorly differentiated squamous cell carcinoma

## Discussion

Basaloid squamous cell carcinoma of the lung as a variant of squamous cell carcinoma is mostly observed in the proximal bronchi. Most of the recent clinical research studies have generally focused on conducting survival analysis of basaloid carcinoma (BC) and poorly differentiated squamous cell carcinomas (PDSC). Moreover, a study conducted by Moro-Sibilot et al. in 2008 examined the prognosis and difference in survival rates between the variant of squamous cell carcinoma and large cell carcinoma [6]. However, the clinical features and prognosis of basaloid squamous cell carcinoma of the lung remained largely unexplored. Hence, this single-center and retrospective study examines and compares the clinical features and prognosis of basaloid squamous cell carcinoma with PDSC of the lung to determine whether the former requires special clinical treatment.

This study found that there were no significant differences between basaloid squamous cell carcinoma and PDSC cases in the proportion of patients who smoked (p = 0.513). This study also showed that the proportion of male was significantly higher among the PDSC cases (p = 0.001), and the proportion of male who smoke was significantly higher among the basaloid squamous cell carcinoma cases (p = 0.003). These findings differ from other studies such as the one by Moro-Sibilot [6] which showed that tobacco consumption was significantly higher among BC patients compared to non-basaloid carcinoma patients. Also, it was found that the age of BC patients was older than non-basaloid carcinoma patients (65.8 vs. 62.4, *p *= 0.03). In addition, a study conducted by Kim et al. in 2003 concluded that there were no significant differences in the clinical and biological features of BC and PDSC patients in Yonsei University Hospital including age, gender, smoking history, pulmonary function and the location of tumors [4].

The results of this study demonstrated that the median survival time of basaloid squamous cell carcinoma and PDSC patients were 19 months and 42 months respectively-there was no significant difference in survival time between the basaloid squamous cell carcinoma and PDSC groups (χ^2 ^= 1.200, *p *= 0.273). There were also no significant differences in the survival rate of patients in stage I and II between the two groups. These findings suggest that the same therapeutic modes can be administered to basaloid squamous cell carcinoma and PDSC patients.

In contrast to this study's findings, a study conducted by Moro et al. in 1994 showed that for patients in stage I and II, there was a significant difference in the 5 year survival rate between BC and PDSC groups-15% and 47% respectively, as well as the median survival time of 606 and 1218 days respectively (p = 0.009) [7]. However, no significant differences in median survival time of patients in stage III and IV were found between the two groups [7]. Kim et al's study found no significant differences between the prognosis of BC and PDSC groups [8]. The 5-year survival rate of all patients between the BC and PDSC groups was 36.5% and 40.6% (p = 0.86), and median survival time was 34.4 months and 34.0 months respectively. The 5-year survival rate of patients in stage I and II between the BC and PDSC groups was 53.9% and 57.7% (p = 0.97) respectively. Additionally, there were no significant differences in relapse rate between the two groups (p = 0.584). Further analysis demonstrated that among patients in stage I, no lymph node metastasis occurred, and there were no statistical differences in the 5-year survival rate between BC and PDSC groups-71.8% and 62.1% respectively, as well as the median survival times of 79.6 months and 106.7 months respectively (p = 0.79).

The findings of this study showed that prognosis is significantly influenced by treatment mode and clinical stage of the tumor. The post-operation mortality hazard of patients treated with a combination of chemotherapy and radiotherapy was 1.296 times higher than other treatment modes (p = 0.025). The post-operation mortality hazard of patients in stage III and IV was 2.035 times higher than patients in stage I (p = 0.047). Additionally, there was no significant difference in post-operation mortality hazard between patients in stage I and stage II (hazard ratio = 2.006, p = 0.069). These results differ slightly from other research such as Kim et al.'s study, which revealed that age (60 years old, hazard ratio = 2.179, p = 0.000) and an advanced clinical stage (stage III, hazard ratio = 2.264, p = 0.000) significantly influenced BC prognosis [7]. Additionally, Coppola et al. found that prognosis was related to the proportion of basal cells in tumors-higher basal cell counts were associated with poor prognosis [1]. Also, Moro et al.'s study demonstrated that operative type (i.e. pneumonectomy, lobectomy, segmentectomy) has no bearing on the prognosis of BC and PDSC patients [8].

Differences between the findings in this study and other research may be due to variations in the source of case subjects, treatment modalities, environmental factors and research design. For example, retrospective analysis was conducted in this study; however, most of the other studies did not conduct retrospective analysis. Additionally, analyses on prognosis and factors influencing prognosis were based on basaloid squamous cell carcinoma patients, in contrast, most of the other studies based their analyses on patients with BC or large cell carcinoma.

## Conclusions

Although there is a low incidence of basaloid squamous cell carcinoma of the lung, it remains a high degree malignancy tumor that is difficult to diagnose before surgery. In addition, there is no agreed upon treatment for patients with basaloid squamous cell carcinoma. This study demonstrated that basaloid squamous cell carcinoma and PDSC have very similar clinical features, and there are no significant differences in survival rates between the two groups. Hence, we conclude that in the short term, the same clinical treatments and therapeutic modes can be administered to patients with basaloid squamous cell carcinoma and PDSC of the lung. Further research on a larger scale needs to be conducted to confirm this conclusion in the long run.

## List of abbreviations

BC: Basaloid carcinomaa; IASLC: International Association for the Study of Lung Cancer; PDSC: Poorly differentiated basaloid squamous cell carcinoma; SPSS11.0: Statistical Package for the Social Sciences; WHO: World Health Organization

## Competing interests

The authors declare that they have no competing interests.

## Authors' contributions

LZ, CLW and LW participated in the design of the study and coordination, KS and JAW helped to draft and modified the manuscript, QLW participated in the data collect and modified the manuscript, HZ performed the statistical analysis, ZLZ and LNS carried out the analysis of the pathological sections. All authors read and approved the final manuscript.
